# Comparison of fluid absorption during transurethral resection of prostate and Holmium-Yag laser enucleation of benign adenoma of prostate using breath ethanol concentration

**DOI:** 10.4103/0970-1591.32061

**Published:** 2007

**Authors:** Shivadeo Bapat, Salil Umranikar, Vikram Satav, Abhijeet Bapat, Arun Joshi, Gauri Ranade

**Affiliations:** Dr. Y. G. Bodhe Department of Urology, Maharashatra Medical Foundation's Ratna Memorial Hospital, S. Bapat Road, Pune, India; * Dr. Y. G. Bodhe Department of Anesthesiology, Maharashatra Medical Foundation's Ratna Memorial Hospital, S. Bapat Road, Pune, India

**Keywords:** Breath ethanol concentration, fluid absorption, Holmium laser enucleation of the prostate, transurethral resection of the prostate

## Abstract

**Objective:**

We conducted a study to detect, quantify and compare irrigation fluid absorption in transurethral resection of the prostate (TURP) and Holmium laser enucleation of the prostate (HoLEP), using BEC.

**Materials and Methods:**

The study included 50 patients of lower urinary tract symptoms, secondary to benign enlargement of prostate. The patients were nonrandomly allocated to undergo TURP and HoLEP. Twenty-six patients underwent TURP and the remaining 24 underwent HoLEP. Sterile water tagged with 1% ethanol w/v was used for irrigation. Absorption was detected and quantified every 10min by BEC levels. Data was analyzed using standard nomograms.

**Results:**

In HoLEP, 14/24 had no fluid absorption. The remaining 10/24 showed fluid absorption ranging from 95 ml to 300 ml. In TURP, all had fluid absorption ranging from 250-980 ml. Three TURP patients developed overt symptoms, while none did in the HoLEP group.

**Conclusions:**

Fluid absorption observed in our study in the HoLEP group was lower than in the TURP group.

Transurethral resection of the prostate (TURP), as of today, remains the ‘gold standard’ and the commonest modality for the surgical management of benign enlargement of prostate (BEP). But it is still associated with significant morbidity (18%).[1] Bleeding and fluid absorption leading to transurethral resection (TUR) syndrome are important and at times, life-threatening. Reported incidence of TUR syndrome is variable and ranges from 0.5-7%.[2] The syndrome occurs due to absorption of irrigating fluid, both in the intravascular and extravascular compartments and manifests with a variety of signs and symptoms secondary to a number of pathologic systemic changes. Holmium laser enucleation of the prostate (HoLEP) is a newer modality in the urological armamentarium for the surgical treatment of BEP. This must however be compared to the existing gold standard both intraoperatively and in its long-term efficacy. The present prospective study was conducted in an attempt to compare irrigating fluid absorption and the risk of the TUR syndrome between these two modalities, using well-established techniques of breath ethanol concentration (BEC). This is the first study of its kind in Indian patients.

## MATERIALS AND METHODS

The study was conducted on 50 patients who had lower urinary tract symptoms (LUTS) secondary to BEP and needed surgical intervention. The age ranged from 51 to 90 years, with a mean of 63.5 years. The patients were non-randomly allocated to undergo TURP and HoLEP. The Institutional Ethics committee approved the study. Informed consent was taken from all patients. The exclusion criteria were prior history of ethanol abuse or habitual intake of alcohol, significant cardiovascular, hepatic, renal or psychiatric disorders, ASA Grade 3 or 4 and debilitation due to pulmonary diseases. After thorough history and physical examination all were investigated as per the institutional protocol. Ten out of 50 patients were operated under spinal anesthesia, while the remaining 40 had epidural anesthesia. Intravenous fluid supplementation consisted of Isolyte M, 5% dextrose solution and Ringer's Lactate solution. Diabetic patients were covered with added insulin. Postoperative supplementation consisted of 0.9% sodium chloride. The surgical intervention was carried out with a conventional continuous irrigating 26 Fr Storz resectoscope using electocautery in 26 patients and Sphinx Lisa Holmium YAG laser with power setting of 80W in 24 patients. The height of the fluid reservoir was kept at 60 cm above the symphysis pubis. No intraoperative suprapubic drainage or diuretics were used. The irrigant fluid used was sterile autoclaved water, tagged with 1% ethanol w/v. sodium chloride is a preferred solution for HoLEP, while glycine is preferred for TURP as an irrigating solution. To maintain uniformity in the two groups, we chose sterile distilled water in both groups. Intraoperative monitoring was carried out at 10 min intervals and included blood ethanol levels, heart rate, electrocardiogram, blood pressure, SpO2, mental status and other signs and symptoms of the TUR syndrome.

**Figure 1:**
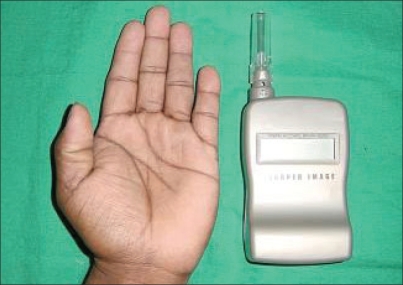
“Digital breathalizer” (Sharper Image - USA)

**Figure 2:**
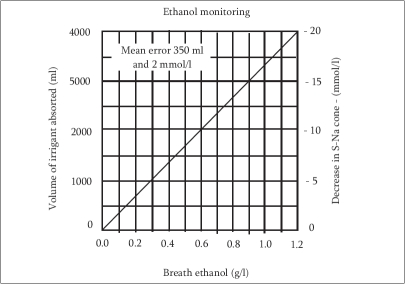
BEC Nomogram

Blood ethanol levels were measured with a “Digital breathalizer” (Sharper Image - USA) which measures the alcohol content in the exhaled breath and extrapolates the value to the corresponding blood level [Figure 1]. The patients were instructed to inhale and then exhale into the mouthpiece of the device every 10 min. The data was tabulated and analyzed using a standard nomogram [Figure 2]. Mean preoperative prostate volume by transabdominal ultrasound was 39.5g (range, 28-66g) for the HoLEP group and 42.2g (range, 33-79g) for the TURP group [Table 1]. The average resection time between the TURP (43.5 min) group and HoLEP (40 min) group and mean total volume of irrigant fluid used (HoLEP 16.91, TURP 17.88 liters), were similar. The mean resected volume (HoLEP 13.8, TURP 19.7g) varied due to partial vaporization of tissue in HoLEP [Table 2].

**Table 1:** Preoperative patient data

	Mean	Range
Age	63.5 years	51-90
International prostate symptom score	21	15-28
Preoperative gland volume HoLEP	39.5 gms	28-66 gms
Preoperative gland volume TURP	42.2 gms	33-79 gms

**Table 2:** Results

Patient data	Holmium laser enucleation of the prostate	Transurethral resection of the prostate
Gland resected (mean)	13.8 grams	19.7 grams
Resection time (mean)	42.2 mins	43.57 mins
Irrigation fluid used (mean)	16.91 lits	17.88 lits
Average fluid absorption	95.8 ml	257.6 ml
Maximal fluid absorption	33 ml	980 ml

## RESULTS

In this study, fluid absorption was detected in 32 / 50 patients. Of the 18 patients who showed no fluid absorption, 14 had undergone HoLEP. Out of the 32 patients who showed fluid absorption, in 29 patients the volume of absorption was between 100- 499 ml. In the remaining three patients, there was significant absorption measuring between 500-1000 ml. These three patients became restless on the table and had bradycardia (heart rate < 50/min) and hypertension. They also complained of headache and were mildly tachypnoeic. There were no visual disturbances or excessive drowsiness in any of these patients. All three had undergone conventional TURP.

Fluid absorption between the two groups showed significant differences. While HoLEP showed average and maximal fluid absorption of 95.8 ml and 300 ml respectively, the same figures were 257.6 ml and 980 ml in TURP group [Tables 2 and 3]. Noticeably, 14/24 patients of the HoLEP group showed negligible (< 150 ml) absorption, which can be attributed to the unique biophysical properties of the Holmium-YAG laser. Comparison between the two groups on various parameters revealed that the fluid absorption during HoLEP is minimal as compared to standard TURP. Postoperatively all patients fared well at three months follow-up.

**Table 3:** Fluid absorption as per breath ethanol concentration levels

Fluid absorbed as per breath ethanol concentration levels (ml)	Transurethral resection of the prostate	Holmium laser enucleation of the prostate
<150	04	14
150-249	07	04
250-299	07	02
300-499	05	04
500-899	01	–
>900	02	-

## DISCUSSION

TURP, as of today, is the commonest surgical procedure for the management of BEP and is considered as the ‘gold standard’ against which all other alternatives are being compared.[1] However, it is not without its risks, including a morbidity of 18%.[1] TUR syndrome was first described by Creevy in 1947, as water intoxication causing hemolytic jaundice and acute tubular necrosis.[3] The reported incidence varies between 0.5-10 %[1–4] in some series. Clinically, it manifests with a variety of signs and symptoms like mental confusion, nausea, vomiting, hypertension, bradycardia and visual disturbances. The etiology and pathogenesis has now been well documented and occurs due to the absorption of irrigating fluid during resection. There are many components which play a role including dilutional hyponatremia, hypervolemia, dyselectrolytemias, hypo-osmolality, renal dysfunction due to glycine toxicity and associated factors like bacteremia and patient cooling. A severe TUR syndrome may eventually lead to coma and death. The degree and rate of absorption depend on a number of factors such as the volume of resection, total resection time, nature of fluid used, height of the reservoir and technique used. Absorption occurs through both prostatic veins (intravascular) and by extravasation (extravascular) routes. While extravascular absorption tends to be more protracted, intravascular absorption is associated with alarmingly rapid uptake, with symptoms developing earlier and are more pronounced.[6] In HoLEP, rapid absorption of the energy into water within the surrounding tissues ensures that the depth of penetration does not exceed 0.5-1 mm, with ablation occurring by a predominantly vaporizing effect.[7] This is the predominant effect on the prostatic tissue. Further, cutting and coagulation occur at the same time and thus smaller blood vessels are sealed off instantaneously, decreasing blood loss and intravascular absorption. Fluid dynamics associated with absorption show two distinct phases.[8] The first phase is characterized by hypervolemia, marked diffusion of sodium and potassium ions from the interstitium into the circulation and elevation of the CVP. This is clinically manifested as bradycardia and hypertension and develops after about 20 min of absorption. The second phase occurs with absorption exceeding 35-40 min and is characterized by progressive hypotension, depressed CVP and hyperkalemia. Uptake in a given individual is haphazard and cannot be predicted. Therefore, absorption needs to be monitored to allow for early intervention of vascular overload, to prevent development of overt symptoms.

There are a number of monitoring techniques available such as volumetric,[9] gravimetric,[9,10] serum sodium dilution,[11–13] breath ethanol test,[8,14–17] serum acid phosphatase[18] and radioisotope method.[9,19] The ethanol breath test was chosen because it is fairly accurate, inexpensive in comparison and user-friendly.[10] Moreover, readings are not affected by spillage of irrigating fluid and administration of intravenous infusions, because of rapid and equal distribution of ethanol in all body compartments. Lastly, blood loss and pulmonary disease do not affect results and alcohol intoxication has not been observed at concentrations of 1% (w/v).

The three patients in the TURP group in our series who had significant fluid absorption and TUR syndrome, had a mean preoperative gland volume of 66 g and mean resection time of 70 min. They were operated by the same surgeon and there was no obvious capsular perforation during the procedure. All three patients were administered Furosemide intraoperatively on completion of the TURP, when fluid absorption was suspected. Postoperatively they were given intravenous 3% sodium chloride solution, till symptomatic improvement occurred. Serum electrolytes were repeated every 4h, till the patients stabilized. All patients were managed conservatively.

Today, there are a number of minimally invasive modalities for the surgical ablation of the prostate. The introduction of laser was greeted with much anticipation by urologists. Although a number of lasers are available, the Holmium YAG laser is the most versatile. It differs significantly from those that preceded it. With a pulse duration of 250 to 350 m, the vapor bubble is pear-shaped and collapses asymmetrically with weak cavitation and minimal plasma formation producing a primarily photothermal effect.[20–22] A number of studies done have shown advantages of laser over conventional TURP including decreased postoperative irrigation, less postoperative catheter time, shorter hospital stay, less hematuria, and discomfort.[23,24] No studies however are available, comparing fluid absorption between these two modalities to the best of our knowledge.

## CONCLUSION

Fluid absorption is definitely less with HoLEP as compared with standard TURP.
